# The Dynamics of Transmission and Spatial Distribution of Malaria in Riverside Areas of Porto Velho, Rondônia, in the Amazon Region of Brazil

**DOI:** 10.1371/journal.pone.0009245

**Published:** 2010-02-16

**Authors:** Tony Hiroshi Katsuragawa, Luiz Herman Soares Gil, Mauro Shugiro Tada, Alexandre de Almeida e Silva, Joana D'Arc Neves Costa, Maisa da Silva Araújo, Ana Lúcia Escobar, Luiz Hildebrando Pereira da Silva

**Affiliations:** 1 Laboratory of Epidemiology, Instituto de Pesquisas em Patologias Tropicais, Porto Velho, Rondonia, Brazil; 2 Laboratory of Entomology, Instituto de Pesquisas em Patologias Tropicais, Porto Velho, Rondonia, Brazil; 3 Laboratory of Molecular Parasitology, Instituto de Pesquisas em Patologias Tropicais, Porto Velho, Rondonia, Brazil; 4 Research Center of Tropical Medicine, Porto Velho, Rondônia, Brazil; 5 Department of Biology, Núcleo de Ciências e Tecnologia, Federal University of Rondônia, Porto Velho, Rondônia, Brazil; 6 Department of Medicine, Núcleo de Saúde, Federal University of Rondônia, Porto Velho, Rondônia, Brazil; University of Liverpool, United Kingdom

## Abstract

The study area in Rondônia was the site of extensive malaria epidemic outbreaks in the 19^th^ and 20^th^ centuries related to environmental impacts, with large immigration flows. The present work analyzes the transmission dynamics of malaria in these areas to propose measures for avoiding epidemic outbreaks due to the construction of two Hydroelectric Power Plants. A population based baseline demographic census and a malaria prevalence follow up were performed in two river side localities in the suburbs of Porto Velho city and in its rural vicinity. The quantification and nature of malaria parasites in clinical patients and asymptomatic parasite carriers were performed using microscopic and Real Time PCR methodologies. *Anopheles* densities and their seasonal variation were done by monthly captures for defining HBR (hourly biting rate) values. Main results: (i) malaria among residents show the riverside profile, with population at risk represented by children and young adults; (ii) asymptomatic vivax and falciparum malaria parasite carriers correspond to around 15% of adults living in the area; (iii) vivax malaria relapses were responsible for 30% of clinical cases; (iv) malaria risk for the residents was evaluated as 20–25% for vivax and 5–7% for falciparum malaria; (v) anopheline densities shown outdoors HBR values 5 to 10 fold higher than indoors and reach 10.000 bites/person/year; (vi) very high incidence observed in one of the surveyed localities was explained by a micro epidemic outbreak affecting visitors and temporary residents. Temporary residents living in tents or shacks are accessible to outdoors transmission. Seasonal fishermen were the main group at risk in the study and were responsible for a 2.6 fold increase in the malaria incidence in the locality. This situation illustrates the danger of extensive epidemic outbreaks when thousands of workers and secondary immigrant population will arrive attracted by opportunities opened by the Hydroelectric Power Plants constructions.

## Introduction

The Porto Velho Municipality, capital of Rondônia State, in Western Brazilian Amazon, has a surface area of 35,000 square kilometers and 386,000 inhabitants [1]. This corresponds to less than 1% of the area and 2% of the total populations of the Amazon region of Brazil. Nevertheless this Municipality has been responsible, in the last years, for more than 6% of the total malaria cases of the all region. Moreover, Porto Velho has been the site of large malaria outbreaks in the course of the 19^th^ and 20^th^ century with thousands of deaths [2]. The two largest epidemic outbreaks occurred in the 20^th^ century, during the construction of the Madeira-Mamoré railroad in 1910 and the second in the 1970–90 colonization decades, after opening of roads to Rondônia from the Southern Central and Northeastern States of Brazil. Two main factors were associated to both outbreaks: (i) a massive immigration of foreign workers or Brazilian workers from regions were malaria had been eradicated, (ii) an intensive environmental impact that followed the deforestation, with degradation of small tributary rivers courses, locally called “igarapés”, resulting in temporary or permanent large water collections, favoring the proliferation of *Anopheles darlingi* the local important vector.

Socio-demographic and epidemiological studies, performed during the 1980's colonization process, defined malaria profile as “frontier malaria” [3], [4]. “Frontier malaria” affected the recently established rural populations, with very high malaria incidences (Annual Parasite Incidence - “API” [5], greater than 1,000 cases per 1,000 inhabitants), with predominance of *falciparum* over *vivax* malaria. The population at risk consisted mainly of adult men, because of their occupational exposure to the mosquito bites.

In the last decades, the incidence of “frontier malaria” has decreased, occurring only in a few new agricultural settlements promoted by the Federal Government or by land invasion due to poverty. The original rural settlements evolved either into stable productive little farms or fused forming large cattle breeding farms. In both situations improvements in housing and basic Public Health resulted in a residual hypoendemic malaria situation with large dominance of *vivax* over *falciparum* malaria.

Research Programs, developed in the last ten years, identified two types of residual malaria in Porto Velho and neighboring Municipalities of Rondônia, associated to the rain seasonality. The rainy season extends from October to May with a total annual precipitation of 2,000 to 2,5000 mm: (i) dry-land malaria, observed in stable agricultural settlements, productive farms, and poor areas in the periphery of cities, show seasonal peaks of incidence following the rainy periods [6], [7], [8], [9]; (ii) riverside malaria observed in riverside areas of the Madeira River and its large tributaries. Peaks of malaria occurred at the height of the rainy season, mainly in children and young adults of either sex [2], [10], [11], [12]. The high Anopheline densities, observed in riverside areas, are often related to seasonal agriculture, the creation of fish breeding tanks, cattle feeding, open gold mining activities and the construction of roads and bridges. These activities promoted artificial temporary or permanent water collections that are excellent breeding sites of *An. darlingi*, the most important malaria vector identified in this region [13], [14], [15], [16], [17], [18], [20], [21], [22], [23]. *An. darlingi* is rarely found in forests [19].

Human bite rates by Anopheline mosquitoes in riverside areas can reach 100, 300 or more bites/person/night [24], [25]. This results in the development of natural immunity among the residents living a long time in this area. Asymptomatic carriers of malaria parasites are therefore frequent in these areas and infect *Anopheles* [10]. The presence of asymptomatic malaria parasite carriers explains earlier malaria outbreaks among rubber exploiters (“seringueiros”) and non-immune workers of the previous migratory flows [2], [3], [4,].

**Figure 1:**
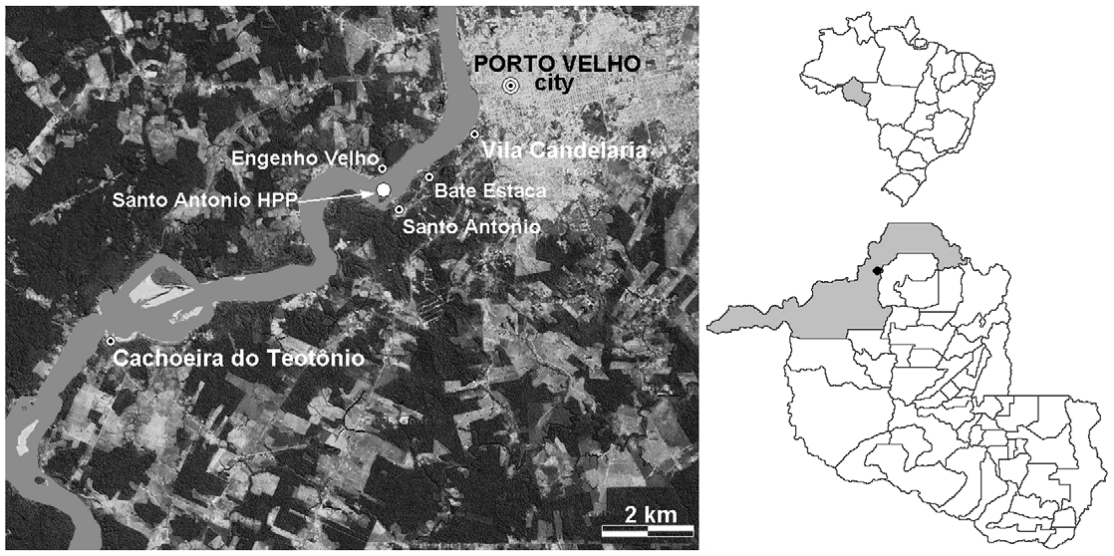
Porto Velho City and a general view of the Madeira River valley. Location of the Porto Velho Municipality, Porto Velho City and a general view of the Madeira River valley, showing the sites of the future construction of the Hydroelectric Power Plants in Santo Antônio and Jirau. Riverside areas of the Madeira River to the south of Porto Velho City, showing localities that will suffer environmental and demographic impacts with the construction of the Hydroelectric Power Plant in Santo Antônio.

The construction in the near future of two Hydroelectric Power Plants (HPP), one in Santo Antônio, close to the city of Porto Velho is expected to have impact on the malaria incidence of the riverside areas of the Madeira River (Figure 1). Various small peri-urban and rural riverside localities in the vicinity of Santo Antônio, have a high incidence of *vivax* and *falciparum* malaria, with API values of 300–1,300 [5], [11], [26], [27]. These localities also present a high prevalence of asymptomatic carriers of malaria parasites [11], [27].

An important flow of immigrants towards these areas is expected to occur as a result of the construction of the HPP, i.e., workers to be recruited and their families and secondary populations attracted by new job opportunities. These workers will probably becoming mostly from non-riverside areas of Porto Velho and neighbor municipalities of Rondônia or neighboring States. Most of them will not have had any prior malaria exposure.

No plans have been made for their future installations settling them in improvised provisory construction likely to be in the vicinity of riverside localities, very exposed to mosquitoes. To avoid the occurrence of new malaria outbreaks in these areas it will therefore be necessary to either improve methods to decrease the parasite sources of infection by active search and treatment of symptomatic and (eventually) asymptomatic parasite carriers, or protect the non-immune workers from contact with parasites sources or both.

As a first control measure we propose by means of the present manuscript to analyze the current dynamics of transmission and occurrence of malaria in two riverside localities in this area. Both have high occurrence of malaria and its inhabitants will be directly exposed to the impact of the two HPP in the near future. These two sites are peri-urban locality of Vila Candelária and the rural locality of Cachoeira do Teotônio.

## Materials and Methods

### Study Area

**Figure 2:**
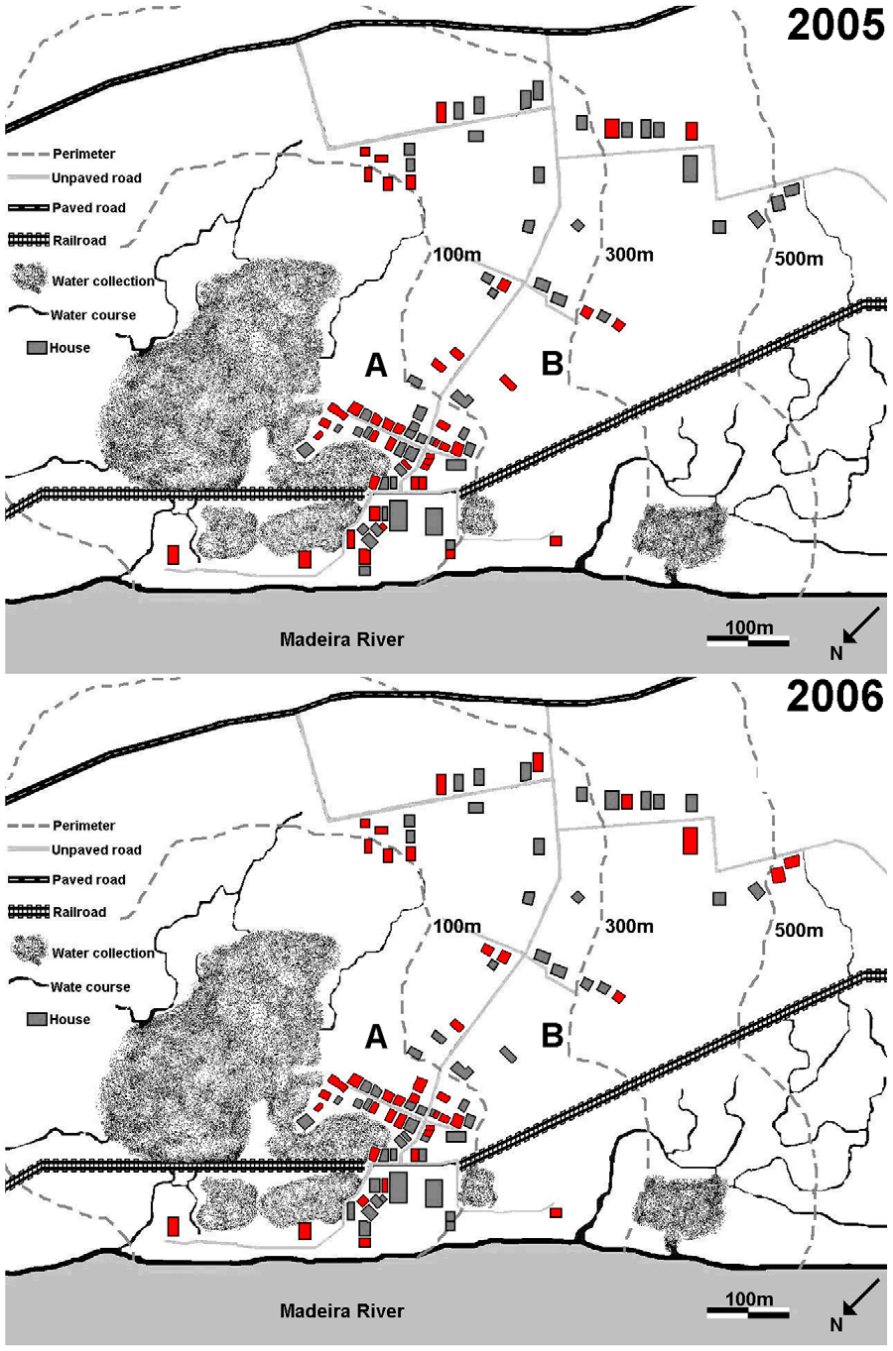
Candelária map representation in 2005 and 2006. Candelária household's representation (little squares). Sub area A contains households up to 100 m distance of mosquitoes' breeding sites (dark spots). Sub area B contains households more than 100 m distant of the breeding sites. Red colors represent houses with malaria cases (2005 and 2006).

The study was performed in two localities, namely Vila Candelária (Candelária) and Cachoeira do Teotônio (Teotônio). Both localities are situated on the right-banks of the Madeira River in the Porto Velho Municipality (Figure 1). They had been previously described in detail [11], [27]. Briefly, Candelária is located at the southern limit of the urban area of Porto Velho city. It is a poor middle class neighborhood, with 370 inhabitants in 2005, and 335 in 2006, with a total of 94 houses, from which only 90 and 81 were permanently occupied in 2005 and 2006 respectively. All these houses have electricity, water supply and basic sanitation. In Figure 2, the households of Candelária are schematically distributed with the distances to one of the two important mosquito breeding sites of this locality.

**Figure 3:**
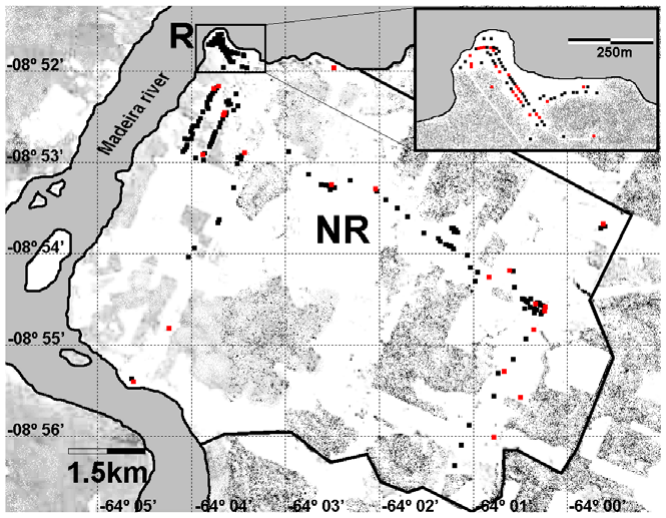
Teotônio map representation in 2007. Teotônio household's representation (little squares). R  =  Riverside area with households of “riberinhos” (amplified in the right upper angle); NR  =  Non riverside area with households of “non ribeirinhos”. Red colors represent houses with malaria cases (2007).

The rural locality of Teotônio is located at 35 km from the city center with access to a paved road and regular daily bus service. It had 379 inhabitants in 2007, divided in two groups: a first group of 50 households with 160 inhabitants, built 50 m from the riverbank. The economic activities of the people living in this area are subsistence agriculture and fishing. The majority of the inhabitants is long time residents of this area and is considered to be true “ribeirinhos” (riverside living people). This locality attracts a multitude of professional and amateur fishermen, particularly during the season of fish reproduction (“piracema”) from May to June. The second group of Teotônios' dwellings consisted in 2007 of 68 households with 219 inhabitants, located in dry land, away from the riverbanks (Figure 3). These houses were constructed along the non-paved road, which runs from the riverbank towards the inner areas covering approximately 45 km^2^. This road is lined by small cattle raising farms and agriculture of subsistence. Most dwellers of this area arrived relatively recently from other parts of the State of Rondônia. They are not considered riverside living people “non ribeirinhos”. Their houses have electricity but no water supply nor sanitation.

### Study Design

The study was based on combined demographic, clinical, parasitological, epidemiological and entomological analyses. After a preliminary demographic census of the study areas a clinical and parasitological cross-section survey was performed to evaluate malaria prevalence. All dwelling were located by a global positioning system device Garmin eTrex Vista™. In order to characterize the place of residents we created a continuous co-variant system of housing providing the linear distance between each dwelling and the river-bank. The identity of all residents of each house, their age, sex, and socio-economic status were established as well as their most important health events most particularly associated with malaria morbidity. A rapid physical and clinical examination was performed by a physician with measure of body temperature and search of signs like liver and spleen enlargement.

For the malaria prevalence survey, performed just after the demographic census, 5 ml of venous blood were collected for the diagnosis of malaria from all residents of both localities, 5 years or olders, regardless of presence of clinical symptoms; 69% of residents of Candelária over 5 years old, and 84% from those of the riverine area of Teotônio accepted to participate and were enrolled. The laboratory diagnosis of malaria was based on microscopic examination of thick blood smears and by nested polymerase chain reaction (PCR) amplification of a species-specific segment of the 18S rRNA gene of human malaria parasites. DNA species PCR amplification was isolated from 200 µl of whole blood using the GFX™ Genomic Blood DNA Purification Kit (Amersham Pharmacia Biotech, Piscataway, NJ). For the nested PCR we used the PCR GeneAmp PCR System 9700 Applied Biosystems Machine and the primers proposed by Snounou et al. [28].

Thick blood smears were stained by Giemsa and examined at a 1,000× magnification of at least 100 fields by two experienced local technicians. All positive slides as well as slides of blood samples collected from febrile individuals and considered to be negative by the local outpost microscopist were sent for re-examination by expert technicians at the Research Center of Tropical Medicine (CEPEM).

The project' procedures were analyzed by the Ethical Research Committee (CEP) of the Research Center of Tropical Medicine (CEP-CEPEM) of the Health Department of Rondônia State Government and considered as “scientifically and technically in accordance with ethical principles of conduct”. Hematological and serological samples of individuals of the study population were collected after informed consent and written agreement of each individual of the population sample. Decision CEP-CEPEM number 25/2004.

### Malaria Surveillance

The baseline survey was continued by a prospective follow-up with the periodic visit (5 times a week) of the inhabitants, asking particularly about febrile episodes followed by active search of malaria parasites in every individual who presented fever or other symptoms of malaria. Persons presenting any suspect signal or symptom of malaria were submitted to clinical and parasitological examination performed by our field team in Candelária starting in 2004 and continued in 2005 and 2006. The study in Teotônio was performed in 2007. This surveillance was maintained between January 2004 to December 2006 for the population of Candelária, and between October 2006 to December 2007 for the population of Teotônio. Households of Candelária were identified according to their distance from identified mosquitoes' breeding sites (Figure 2) of less than 100 m (area A), and more than 100 m (area B). The households of Teotônio (Figure 3) were classified in two groups: “ribeirinhos” (area R), i.e., located along the river-banks and “non-ribeirinhos” (area NR), those located in the inland areas. For the purpose of surveillance, health workers visited the area during five times per week (Monday to Friday) and examined all participants of this study area (irrespective of their ages), reporting current or recent fevers, headaches, or other symptoms of malaria. Venous or finger-prick blood samples were obtained for the diagnosis of malaria by microscopy and PCR. As part of the surveillance, the files of all patients were kept at the two local outposts for malaria diagnosis. Thick blood smears were examined by local microscopists. The DNA templates for PCR amplification were obtained from venous blood samples, as detailed previously [11].

### Malaria Incidence and Definitions

Following the Brazilian Ministry of Health criteria, a malaria case was defined by the presence of clinical symptoms associated to the presence of parasites in the microscopic examination. Official registers of the- SIVEP-Malária, the official agency of the Ministry of Health [5], quantify malaria incidences in a locality by Annual Parasite Incidence (API  =  number of clinical malaria cases/1000 inhabitants). SIVEP-Malária figures were, for Candelária 556 in 2005 and 472 in 2006. These differences were, however non significant (p = 0.888). For Teotônio the value registered in 2007 was 1,865. These API values were calculated in reference to demographic data of the IBGE 2000 census [1] that registered 250 inhabitants for Candelária and 104 inhabitants for Teotônio. In addition, according to SIVEP-Malária criteria, the number of cases registered in a locality is not restricted to clinical malaria episodes registered among the local residents, but is extended to visitors and temporary residents that declare, in the moment of the diagnostic procedure, to have acquired the infection in this locality.

In the present work, in addition to the access to official registers of SIVEP-Malária cases, we performed a demographic census every year and were able to distinguish, in one side, malaria cases among visitors and temporary residents and, on the other side, malaria cases of residents. The populations of permanent residents found in Candelária was 370 dwellers in 2005 and 335 in 2006. For Teotônio we registered 379 inhabitants, with 160 living in the riverside areas of the Madeira River (“ribeirinhos”) and 219 living in the inland areas (non “ribeirinhos”). API values registered by SIVEP-Malária and API values calculated by our data on the real localities' population and clinical cases registered by our services could be compared.

### Malaria Treatment

Patients suffering of vivax or falciparum clinical malaria episodes were treated according to the instructions of the Brazilian Ministry of Health Malaria Treatment Manual [29]. Malaria parasite carriers detected by PCR were considered asymptomatic malaria parasite carriers only if, in 30 days following the baseline survey examination, no symptoms were observed. They were treated if they developed fewer, headache or other malaria symptoms and for this they were submitted daily to intensive surveillance by health agents, according to instructions of Brazilian Ministry of Health.

### Entomological Studies

Mosquitoes captures were performed once monthly from January 2004 to December 2006 in Candelária, and from October 2006 to December 2007 in Teotônio. Simultaneous 12 hr captures were performed indoor and outdoors from 6pm/6am by trained technicians, with participation and supervision by the Authors of this manuscript.

Outdoor sites were chosen at distances of no more than 6 m from the indoor sites. Six trained technicians worked in each group, divided in pairs, one person capturing mosquitoes indoors and the other outdoors. Each capturer worked for 1 hr and then rested for the next hour.

The presidence of the local Ethic Committee CEP-CEPEM had provided accordance with the procedure of mosquito capturing in agreement with the procedures recommended by the “Strategies of protection for the professional technician in charge of mosquitoes captures by the Brazilian Ministry of Health [30], including written and signed information about the risk of acquiring malaria during the captures.” No accidental infections occurred during the three years of these activities. Mosquitoes were captured over the capturers' legs with hand-held aspirators. The average hourly number of mosquitoes captured per person was scored as the Hourly Biting Rate (HBR) [31].

### Identification, Dissection and Determination of Mosquito Infection Rates by Malaria Parasites

The Consoli and Lourenço de Oliveira [32], standard key for the identification of Brazilian *Anopheles* mosquitoes, was used for identification. Mosquitoes were dissected and their salivary glands, midguts, and ovaries were removed for examination of the presence of oocysts (midguts) and sporozoites (salivary glands). Salivary glands and midgets were kept in isopropanol for PCR analysis. The tracheoles of the ovaries were examined for the determination of parity. Mosquito samples from each capture were stored: their carcasses were frozen and stored at −20°C, while the salivary glands and midguts were resuspended in 0.5 ml of a 0.9% NaCl solution and then frozen at −20°C for future genetic studies. The samples included mosquitoes captured in different sites, indoor and outdoor, and at different hours of the night.

### Statistical Analysis

Exploratory analysis. A database was created with SPSS 13.0 Software SigmaStat 2.03 (SPSS Inc., Chicago, Il, USA). Correlations were done by the Spearman Test in Candelária (sectors A and B malaria incidence) and Mann-Whitney test for Teotônio (incidence in residents and non residents). Factorial analysis was used for to identify clusters of number of malaria cases in the houses. The age/sex rate comparison between residents and nom residents in the riverside area was analyzed by chi-square. It was considered significant results with p-value<0.05.

## Results

### Base Line Survey and Malaria Prevalence

The clinical and parasitological base line survey performed by our team, after the preliminary demographic census, permitted to establish the parasite rate index of inhabitants with 5 years of age or more in both localities (Table 1). While the demographic and clinical surveys were able to register basic information as clinical signals and past malaria experience for the residents of all age groups, the parasitological survey enrolled only residents over 5 years of age that accepted to participate in the study, representing 69% of residents with more than 5 years in Candelária and 84% in Teotônio. As shown in Table 1, parasite rates obtained by microscopic examination of blood samples were extremely low, 0.5% for Candelária and 3.4% for Teotônio. When using PCR examination, In Candelária was found rates of 1.9% for *P. falcip*arum and 13.2% for *P. vivax*. In Teotônio the were <1% and 15.0% respectively. In the performed prevalence survey no asymptomatic malaria carriers were found by microscopy, all positive slides found corresponding to symptomatic clinical malaria cases. However, some asymptomatic positive PCR cases developed clinical symptoms in the following weeks of surveillance and were treated.

**Table 1:** Parasite rates observed in permanent residents of Vila Candelária (September 2006) and Teotônio Riverine (October 2006).

Locality	Houses	Inhabitants			PTS			PCR		
		Total	≥5	EXA (%)	Pv (N/%)	Pf (N/%)	Total (N/%)	Pv (N/%)	Pf (N/%)	Total (N/%)
Candelária Sep 2006	81	335	298	205 (67%)	0 (<0.5)	1(0.5%)	1 (0.5%)	23 (11.2%)	4 (1.9%)	27 (13.2%)
Teotônio R Oct 2006	50	160	141	119 (84%)	4 (3.4%)	0 (<0.8%)	4 (3.4%)	18 (15.1%)	0 (<0.8%)	18 (15.1%)

Data on population of the localities have been obtained by the preliminary demographic survey, updated every year of the study. API (annual parasite index  =  annual number of cases/1000 inhabitants). API SIVEP/Malaria  =  data obtained from [26]. Pv - *Plasmodium vivax*; Pf - *Plasmodium falciparum*.

Malaria rates observed in cross-sectional surveillance (September 2006 in Vila Candelária, October 2006 in Teotônio), performed by thick smear and PCR assay.

≥5  =  inhabitants over 5 years old.

EXA  =  inhabitants examined.

PTS  =  positive thick smear.

PCR  =  positive PCR for malaria.

N/%  =  population and percentage with positive malaria assay.

Pv  =  *Plasmodium vivax*.

Pf  =  *Plasmodium falciparum*.

### Malaria Incidence in Vila Candelária and Cachoeira do Teotônio

As explained in Methods, the data on malaria incidence observed in Candelária in 2005 and 2006 and in Teotônio in 2007 were expressed by API, values. The APIs officially registered in recent year by SIVEP-Malária [5] for Candelária and Teotônio suffered from underestimation of their respective populations, based on the IBGE census of 2000 [1] and from the inclusion of malaria cases of non residents that declared to have acquired the infection in these localities. In Table 2 we summarize the real incidence of malaria among residents of both localities, from 2005 to 2007, based on the results of our demographic census and the locally performed longitudinal surveillance of malaria cases. It results of API values for residents in Vila Candelária of 316 (262 for vivax and 54 for falciparum) in 2005 and 277 (235 for vivax and 42 for falciparum) in 2006. API values in Teotonio in 2007 were 412 in the riverine area (334 for vivax and 69 for falciparum) and 160 (123 for vivax and 36 for falciparum) in the non riverine, dry land area. The SIVEP-Malária official data registered much higher values and this will be explained in the following items.

**Table 2:** Vivax and falciparum malaria incidence in residents of Candelária (2005 and 2006), and Teotônio (2007).

Year	Locality	Population	Pv cases	Pf cases	Total cases	API Pv	API Pf	API malaria	API SIVEP/Malária*
2005	Candelária	370	97	20	117	262	54	316	556
2006	Candelária	335	79	14	93	235	42	277	472
2007	Teotônio R	160	55	11	66	344	69	412	-
	Teotônio NR	219	27	8	35	123	36	160	-
	Teotônio Total	379	82	19	101	216	50	266	1,865

Candelária A corresponds to the sub area of the locality less than 100 m distant from the mosquitoes' breeding sites; Candelária B corresponds to the sub area more than 100 m distant from the mosquitoes' breeding sites; Teotônio R corresponds to the river-side area with households of riverside dwellers (“ribeirinhos”); Teotônio NR corresponds to non riverside areas (“non ribeirinhos”).

Data on population of the localities have been obtained by the preliminary demographic census, updated every year of the study.

API  =  Annual Parasite Index; annual number of cases/1,000 inhabitants.

API SIVEP/Malária*  =  data obtained from [26].

Pv  =  *Plasmodium vivax*.

Pf  =  *Plasmodium falciparum*.

Teotônio R  =  Riverine area of Teotônio.

Teotônio NR  =  Non Riverine area of Teotônio.

### Distribution of Malaria Cases in Candelária and Teotônio

Table 3 summarizes the distribution of *vivax* and *falciparum* malaria cases occurred among permanent residents and their houses in both localities from 2005 to 2007. Surprisingly, the 117 malaria cases registered in Candelária in 2005 and the 93 cases of 2006 were not randomly distributed in the occupied households of the locality, in number of 90 in 2005 and 81 in 2006. Malaria occurred in only 42 and 40 houses, respectively. Most of the positive households in 2005 remained positive in 2006 (data not shown). We observed that the number of households with malaria decreased according to their distances from the mosquito breeding sites (Figure 3) The median number of houses with malaria cases in sector A is significantly higher than in sector B (p<0.05). As shown in Table 3, the 53 households of area A, closer to breeding sites, there were 30 positives (60%) in 2005, and 27 (48%) positives in 2006. In the 37 houses of area B, only 12 (32.4%) had malaria cases in 2005, and 13 (39.4%) in 2006. The sector's difference was still more clear when we analyzed the distribution of residents affected by malaria. The number of malaria infected residents in 2005 was 55 (25.0%) of 220 people living in area A, and only 19 (12.7%) of resident living in area B (p<0.05).

**Table 3:** Malaria cases distribution by dwellers and dwellings in Candelária (2005–2006), and Teotônio (2007).

Year	Locality	RES						HOU					
		TOT	WOUT	WITH				TOT	WOUT	WITH			
				Pv	Pf	Pv/Pf	Total			Pv	Pf	Pv/Pf	Total
2005	Candelária A	220	165	43	5	7	55	53	23	19	1	10	30
	Candelária B	150	131	15	2	2	19	37	25	8	1	3	12
	Total	370	296	58	7	9	74	90	48	27	2	13	42
2006	Candelária A	200	156	32	4	8	44	48	21	16	1	10	27
	Candelária B	135	110	24	1	-	25	33	20	11	1	1	13
	Total	335	296	56	5	8	69	81	41	27	2	11	40
2007	Teotônio Riverine	160	111	38	8	3	49	50	26	16	2	6	24
	Teotônio Non Riverine	219	193	18	4	4	26	68	45	15	3	5	23
	Total	379	304	56	12	7	75	118	71	31	5	11	47

Candelária A corresponds to the sub area of the locality less than 100 m distant from the mosquitoes' breeding sites; Candelária B corresponds to the sub area more than 100 m distant from the mosquitoes' breeding sites; Teotônio R corresponds to the riverside area with households of riverside dwellers (“ribeirinhos”); Teotônio NR corresponds to non riverside areas (“non ribeirinhos”).

Candelária A correspond to the sub area of the locality distant less than 100 m from the mosquitoes' breeding sites.

Candelária B correspond to the sub area distant more than 100 m from the breeding sites.

RES  =  residents.

TOT  =  Total.

HOU  =  households.

WOUT  =  without malaria.

WITH  =  with malaria.

Pv  =  only *Plasmodium vivax*.

Pf  =  only *Plasmodium falciparum*.

Pv/Pf  =  *Plasmodium vivax* & *Plasmodium falciparum*.

In both areas, it was observed a positive correlation of number of malaria cases with number of residents in the house (data not shown). However, using factorial analysis it was show that the number of residents per houses accounts only for 72% of clusters. Therefore, others factors area involved in malaria cases concentration as shown in the following items

The distribution of malaria cases in the rural locality of Teotônio in 2007 followed the same profile shown in Candelária. Again, malaria was not distributed randomly in 118 houses of this locality and the 101 cases of malaria which occurred in 2007 were concentrated in 47 households. Once again the distance of the households to the mosquito breeding sites was an important risk factor (Figure 3). In the houses of area R (“ribeirinhos”) malaria occurred in 24 (48.0%) of the 50 households. In the houses of area NR (non riverside) malaria occurred in only 23 (33.8%) of the 68 households of this area (p<0.05). Visits found no evidence of any differences in protective measures taken against mosquito bites in malaria positive compared to negative households. Data shown in Table 3 confirm earlier observations on risk factors associated with malaria transmission represented by close proximity to *Anopheles* breeding sites. Additional factor(s) responsible for the concentration of malaria cases in some houses and inhabitants were not clearly identified. These and other factors that potentially correlate with indoor malaria transmission will be analyzed in Discussion.

### Houses' Concentration of Vivax Clinical Cases

As shown in Table 3, malaria transmission occurred only in some of the households where they concentrate in number of cases. In Table 4 it is shown that the number of vivax malaria cases per house in Candelária and in the riverside area of Teotônio varies from 1 to 7 or more.

**Table 4:** House's concentration of vivax clinical episodes in Candelária and Teotônio.

Year	Locality		VMC							
			1	2	3	4	5	6	≥7	Total
2005	Candelária A and B	Houses	14	11	7	4	-	4	-	40
		Vivax cases	14	22	21	16	-	24	-	97
2006	Candelária A and B	Houses	23	6	4	2	2	1	1	39
		Vivax cases	23	12	12	8	10	6	8	79
2007	Teotônio Riverine	Houses	8	6	5	1	-	1	-	22
		Vivax cases	8	12	15	4	-	6	10	55
	Teotônio Non Riverine	Houses	13	7	-	-	-	-	-	20
		Vivax cases	13	14	-	-	-	-	-	27

Candelária A corresponds to the sub area of the locality less than 100 m distant from the mosquitoes' breeding sites; Candelária B corresponds to the sub area more than 100 m distant from the mosquitoes' breeding sites; Teotônio Riverine corresponds to the riverside area with households of riverside dwellers (“ribeirinhos”); Teotônio Non Riverine correspond to non riverside areas (“non ribeirinhos”).

VMC  =  number of vivax malaria cases.

In consequence, in Candelária, in 2005, 97 vivax malaria cases concentrated in only 40 houses, while 48 houses were negative for malaria cases and in 2006, 79 vivax malaria cases occurred in only 39 houses, while 41 cases had no malaria cases The average number of vivax malaria episodes per positive household was 2.78 in 2005, and 2.32 in 2006. The same profile was observed in the riverside area of Teotônio, with 55 vivax cases in 22 houses (2.7 cases per positive houses) and 26 houses negative for malaria.

### Role of Vivax Relapses

A partial explanation for the multiple numbers of *P. vivax* episodes in the households is possible due to *P. vivax* relapses already observed in earlier studies done in the same area [9]. As shown in Table 5, the multiple cases of vivax malaria cases in a house was frequently dependent of vivax malaria episodes occurring in the same person. In the one year period of observation falciparum clinical attacks were usually unique (just one exception) while in 2005, for instance, 17 residents presented 2 clinical episodes and 6 residents presented 3 vivax clinical episodes

**Table 5:** Distribution of malaria cases in residents of Candelária and Teotônio.

Locality	Area	Year	PAT						NPAT		NEPI		
			1		2		≥3						
			Pv	Pf	Pv	Pf	Pv	Pf	Pv	Pf	Pv	Pf	Total
Candelária	A and B	2005	44	16	17	2	6	-	67	18	97	20	117
	A and B	2006	52	12	10	1	2	-	64	13	79	14	93
Teotônio	Riverine	2007	28	11	12	-	1	-	31	11	55	11	66
	Non Riverine	2007	17	8	5	-	-	-	22	8	27	8	35

Data on population N of the localities have been obtained in the preliminary demographic survey, updated every year of the study. Sample collected for parasitological analysis included residents over 5 years of age, correspond to 61% of total population N and75% of the corresponding N fraction.

PAT  =  number of patients with one or more malaria episodes (1, 2 or ≥3 episodes).

NPAT  =  number of patients with malaria.

NEPI  =  number of malaria episodes.

Pv  =  *Plasmodium vivax*.

Pf  =  *Plasmodium falciparum*.

As it can be see in Table 5, the 97 vivax malaria episodes of Candelária in 2005 occurred in 67 patients and the 79 episodes of 2006 affected 64 patients. In Teotônio 55 episodes occurred in 31 patients. Multiple malaria episodes in Teotônio occurred mostly in riverside areas. Double and triple episodes of *vivax* malaria frequently occurred in the same person so that 14 episodes could be considered to be *P. vivax* relapses. This contrast with falciparum malaria episodes that only exceptionally occurs twice in the same patient

Another element that indicate that most multiple vivax clinical episodes in the same patient area dependent of relapses and not of re infections is that multiple vivax episodes registered in Candelária, in 2005 and 2006 occurred in the same house and frequently in the same person (data not shown).

### Malaria Risk in Riverside Madeira River Localities

If all the multiple vivax clinical attacks in the same patients were considered relapses, it would reduce the 97 *vivax* episodes in Candelária of 2005 to 67 vivax episodes produced by original infections (97−30 = 67), and to 64 in 2006 (79−15 = 64), representing the number of original infections given rise to single or multiple clinical episodes of malaria. This would reflect the real *vivax* malaria transmission risk. In this case, the risks of transmission would be evaluated in 18.1% and 5.4%, for *P. vivax* and *P. falciparum* respectively in 2005, and 19.1% and 4.1% in 2006. However, this will be discussed in the next section.

### Seasonal Malaria Incidence and Anopheline Densities

**Figure 4:**
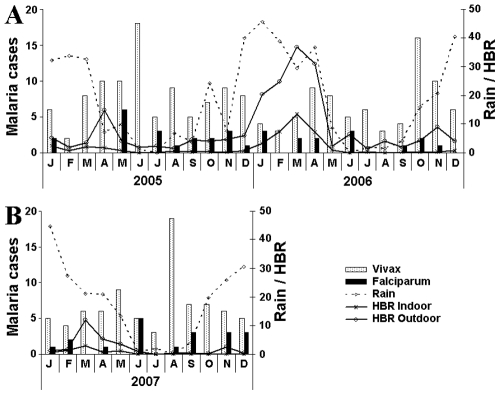
Vivax and falciparum malaria cases monthly distribution. Monthly distribution (histograms) of vivax and falciparum malaria cases in Vila Candelária (A) and Cachoeira de Teotônio (B), in relation to variation of HRB (hour bitting rates), and rain precipitation in Vila Candelária (2005–2006) and Cachoeira do Teotônio (2007).

Figure 4 summarizes the seasonal incidence of *P. vivax* and *P. falciparum* malaria in Candelária (Figure 4A) and Teotônio (Figure 4B), as well as the density of Anophelines, expresses in hourly biting rate (HBR). Rainy periods during 2005, 2006 and 2007 are shown. They occurred regularly between September and May with a total precipitation of 1,989 mm, 2,438 mm and 2,101 mm respectively from 2005 to 2007.

Malaria infections occurred during all these years, increasing from February to May-June for riverside malaria, after the peak of the rainy season. The incidence of malaria decreased during the dry season from June-August. An additional peak of malaria occurred in September-October, at the beginning of the new rainy season.

Figure 4 shows the evolution of monthly averages of human biting rates in the localities. As mentioned previously, the outdoors HRB values are 5–10 folds greater than indoors. The number of annual bites per person was estimated to have been 2,000–10,000 indoor and 10,000–50,000 outdoor. Surprisingly, considering the high prevalence of malaria in both localities, the number found of infected mosquitoes was very low (<0.5%). More surprising was the absence of mosquitoes harboring sporozoites of malaria parasites resulting in the absence of significance Entomological Inoculation Rate (EIR). In the absence of a significant EIR, even considering the oocyst positivity, in other words, a very low transmission rate.

### Micro-Epidemic Malaria Outbreaks

As shown in previous items, the residents of riverside areas of Candelária and Teotônio, have a certain risk of acquiring malaria, i.e. 20–25% of *P. vivax* and 5–7% of *P. falciparum*. In consequence, each locality presents about 100 clinical malaria cases per year, with a tendency of decreasing in recent years. The prevalence of asymptomatic malaria carriers in adults varies between 10–20% what contribute to maintain the endemic situation. Close prevalence's were observed in other riverside localities of the Madeira River, upstream or downstream to the HPP areas (Katsuragawa, personnal communication). The presence, in both localities of facilities for the diagnosis and treatment of malaria, results in the occurrence of only rare cases of severe malaria in need of hospitalization, and complete absence of mortality. The situation could therefore be defined as of relative stability. However, this is in contradiction with the official data of SIVEP-Malária [26] that registers high incidence of malaria in Teotônio, with API index of 1,879 and 1,274 in 2005 and 2007 respectively.

**Figure 5:**
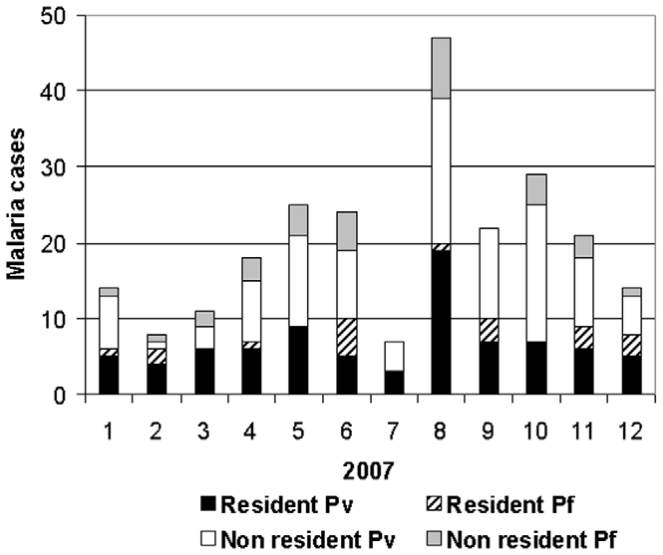
Malaria cases monthly distribution in Teotônio. Monthly distribution of malaria cases in Teotônio among permanent residents (Resident) and temporary residents and visitors (Non resident). Pv  =  *Plasmodium vivax*; Pf  =  *Plasmodium falciparum*.

The numbers proposed by SIVEP-Malária are in fact an over estimation since they were based on an old population census [1] which gives an erroneous number of inhabitants for the two localities. In fact, the demographic census performed by our researcher team in 2007 found 379 residents in Teotônio. The real calculated API would be, after correction, 482.8 in 2005, and 511.8 in 2007. These figures still represent very high incidences, in contrast with much lower API values that we have defined for the permanent residents of the localities, between 266 to 412 (Table 2), that is still high, but much lower. The explanation to re-conciliate these figures for Teotônio is given in Figure 5, where malaria among non residents were introduced showing the monthly number of malaria cases in residents and non residents. A total number of malaria cases of 256 in 2007 (201 were *P.vivax* and 55 of *P. falciparum*) represent, therefore a superposition of malaria of residents (101) and non residents (156).

In our databank malaria incidence among residents could be re-calculated showing a total of 101 cases, 82 being of *P. vivax* and 19 of *falciparum* malaria (Table 2). When comparing the age/sex profile of malaria in the residents of the riverside areas with the non residents a striking difference is observed (Table 6). The profile of patients of the riverside area is typical, with the majority of cases having occurred in children and adolescents of either sex (p<0.05). Among non-residents while a API value cannot be calculated for non residents, since is ignored the number of “non resident” that visited Teotônio or stayed for a period, and did not get malaria infections. However, the age/gender profile of malaria cases is that of “frontier malaria”, with the majority of cases in adult males (p<0.05). Malaria in Teotônio is, therefore, a superposition of two types of malaria: hypoendemic malaria with the riverside profile and epidemic frontier malaria type occurring in non resident fishermen and open gold mine workers.

**Table 6:** Age and genre distribution of patients with malaria in Teotônio (2007).

Locality	Age	Pv		Pf		Total			
		Male	Female	Male	Female	Male	Female	Total	API
Teotonio R	0–4	2	4	-	1	2	5	7	
	5–14	12	9	1	3	13	12	25	
	15–29	10	12	2	1	12	13	25	
	30–50	3	3	-	2	3	5	8	
	>51	-	-	1	-	1	-	1	
	Total	27	28	4	7	31	35	66	412
Teotonio NR	0–4	-	-	1	-	1	-	1	
	5–14	4	4	2	1	6	5	11	
	15–29	1	4	-	1	1	5	6	
	30–50	7	1	1	-	8	1	9	
	>51	6	-	2	-	8	-	8	
	Total	18	9	6	2	24	11	35	166
Non Residents	0–4	2	2	-	-	2	2	4	
	5–14	1	2	1	-	2	2	4	
	15–29	18	9	14	3	32	12	44	
	30–50	16	8	7	4	23	12	35	
	>51	2	3	-	1	2	4	6	
	Total	39	24	22	8	61	32	93	-

Teotônio R  =  correspond to the river-side area with households of riverine dwellers (“ribeirinhos”).

Teotônio NR  =  correspond to non riverine areas (“non ribeirinhos”).

Non residents  =  visitors or temporary residents that declared to have acquired malaria in Teotônio.

Pv - *Plasmodium vivax*.

Pf - *Plasmodium falciparum*.

API–Annual Parasite Index.

This process is repeated every year, since Teotônio is a place preferred by professional and amateur fishermen, particularly during the “piracema”. Fishermen come from the dry-land areas from the vicinity of the city of Porto Velho and from other Municipalities of Rondônia. They spent from few days to few weeks in the area, using camping tents or shacks which remain empty for part of the year. The rudimentary housing makes this temporary population very exposed to outdoor mosquito bites and malaria transmission. This result every year in hundreds of fishermen becoming infected in Teotônio and spreading malaria all over the Municipality of Porto Velho and neighboring localities.

## Discussion

The present investigation of localities of the Madeira river describes the process by which epidemic outbreaks of malaria can and do occur in riverside areas. The presence of asymptomatic malaria carriers, in addition to symptomatic malaria infections, provide an important source of parasites. Combined with the high density of *Anopheles* mosquitoes in the riverside areas they are responsible for malaria outbreaks, particularly due to the arrival of additional population groups. This has been the case in Teotônio, during the period of the present study, in spite of efficient local malaria outpost for diagnosis and treatment. The arrival of hundreds of fishermen, at the end of the rainy season, produces micro epidemic outbreaks that increased the malaria incidence by a 2.6 factor in 2007. It is of interest to point out the age/gender profile of malaria of the local' residents malaria patients that is typical of the riverside malaria, the disease occurring mainly in children and adolescents regardless of their sex. Non-resident individuals present a typical “frontier malaria” profile, male adults being the main population at risk. This situation does not apply exclusively to Teotônio, but occurs to a variable degree to all riverside areas, south of the city of Porto Velho (Figure 2). Statistics of the Ministry of Health showed high incidences, with API values of 500 or more. Previous surveys indicated a high prevalence of asymptomatic malaria carriers, of 10–30% [27].

The arrival of thousands of newcomers in the near future, recruited by the Hydroelectric enterprise, to the riverside areas and the vicinity of Federal Highway BR 364, attracted by secondary jobs underscores the potential for extensive spread of malaria infections.

We will now consider the possible reasons for the high incidence and predominance of *vivax* malaria observed by us, as well as concentration of malaria cases in a fraction of the dwellings of permanent residents of both localities in relation to the vectorial capacity of *An. darlingi*.

We showed that malaria cases among the riverside residents in both localities are not distributed randomly amongst the dwellings, but concentrate in only a fraction of them. If this is suggestive of indoors transmission (since no special protective physical structures were found associated with “negative houses”), the concentration may be dependent on other factors, including some registered in other items of the manuscript such as: (i) the number of inhabitants per house in the positive dwellings; (ii) the relapses of vivax malaria in the “positive houses” considering, for instance, that 97 vivax malaria cases occurred in Vila Candelaria in 2005 in only 67 patients and 79 vivax malaria cases occurred in only 64 patients in 2006 (Tables 4 and 5).

Other facts, however, consistent with indoors transmission were observed both in the present study and described in a previous paper (11) that may be summarized as follows: (i) tropical night starts quite quickly at 6 p.m. and children are soon indoors to be fed and then to sleep; (ii) *An darlingi*, the vector in the area, has a precise period of biting activity, beginning after the twilight (6 p.m. in the area). Female mosquitoes' absent until this time increase quickly to maximum densities at 10 p.m; (iii) children are the main risk group in the riverside malaria area (references 2, 3, 11) and in the particular case of Candelaria the API of children is 510, while that of adults with 40 years of age or more is 110 and this was explained by immunity acquisition in adults (11); (iv) “positive houses” in 2006 were practically the same as in 2005 (see Figure 2A and B); (v) new primary falciparum cases occurred almost exclusively in houses previously positive for vivax malaria cases (Table 3). If the first three elements correlate the children population at risk to indoor transmission, the two other factors suggest that female mosquitoes are attracted to “positive houses”. Experiments performed by our group eliminate the possibility of female mosquitoes returning to the same or nearby house (Gil, personal communication), where they had previously fed. It is however possible that a special attraction stems from “fever episodes” occurring more frequently in houses with more children and more malaria cases.

Since the classical work of Colluzzzi and collaborators [33], a large number of investigators have studied on the molecular identification of mosquito attractants. These efforts concentrated on *Aedes* and *Culex*, but some studies were also done on the African malaria vector, *Anopheles gambiae*. The crystal structure of an odorant-binding protein from *An. gambiae* has also been reported [34]. Furthermore, a synergistic effect of ammonia, lactic and carboxylic acids, all components of human sweat, more actively secreted during fever episodes, has been described in the host-seeking behavior of *A. gambiae* [35].

Our present observations and the factorial analysis of malaria cases clusters in a fraction of the residences suggest that other factors, besides the distance to the mosquitoes' breeding sites and the number of residents in the house contribute to concentration of cases in some of the houses. This could be the sweating due to fever associated with the malaria infection might attract the additional mosquitoes bites. No data are available for *An. darlingi* attractants but they would be of particular interest since there has been no co-evolution, as in Africa, between humans and malaria parasites.

*An. darlingi* has been described as the main vector of malaria in the Amazon region of Brazil. Meanwhile studies on other *Anophele*s species indicate that *An. darlingi* occurs rather rarely in the forests of the Amazon region [19]. Interestingly, this *Anopheles* is consistently the predominant species in malaria endemic areas [22], [23], [24], [36], [37].

Laboratories studies on *An. darlingi* from Rondônia showed its high susceptibility to *vivax* and *falciparum* infections [16]. These studies, however, were done using human volunteers selected in view of their high number of circulating gametocytes (Tada personal communication). Its real vectorial capacity has not been precisely defined in field studies. Most fields studies using human based attraction for mosquitoes' capture indicate five to ten times higher outdoor than indoor densities of *Anopheles darlingi* [22], [24], [37]. This led to the notion that malaria transmission in the Amazon region was mainly due to outdoor mosquito bites. However, when in the same studies, malaria transmission was evaluated by EIR, a surprisingly low rate usually less than 1 was found in areas with API values of 500 to1,000. These numbers contrast with those found for *An. gambiae* in urban, sub-urban and rural areas of Africa. In that case EIR values of 10 that have been reported in urban areas and of 15 to77 in suburbs of African cities [38].

In the present study, we emphasize the possible importance of indoor malaria transmission for the stable resident of the riverside localities, a possibility that needs further investigation. Outdoor transmission seems to be important only for temporary residents or visitors such as fishermen living in camping tents or provisory shacks. Evidently night shift workers will be at risk of outdoor malaria transmission. In view of these observations new control measures of malaria transmission must be introduced in order to pursue two goals: (i) reduction or elimination of the source of infection; (ii) protection of the population at risk.

The first objective must be the central goal of the new control program and must be developed taken into consideration the characteristics of endemic riverside malaria, using a permanent surveillance of the localities, demographic control of the population by Health Agents. This implicate in active search of malaria infections. The observed high prevalence of asymptomatic malaria carriers in the area, as possible source of infections, would indicate also the interest in their treatment. However, to be done, this needs a considerably increase in qualified personnel and investments in new equipments for systematic use of PCR for diagnosis and engagements for continuity of the intervention. Recent work by Aponte et al [39], has shown that the treatment of asymptomatic malaria parasite carriers resulted in a reduction of the disease during the first year of life, but that “after discontinuing the intervention, there was a significant increase in the incidence of clinical malaria for 2 years”. However, it must be considered that all the sub urban and rural areas of Porto Velho municipality under study correspond to the area of impact by the construction of Hydroelectric Power Plans. The project has received important financial funds, from the Government and from the private enterprises in charge of the constructions, including for permanent investment in public health This would create a favorable opportunity to evaluate the impact of the treatment of asymptomatic carriers as well as the chemoprophylaxis of relapses of *P. vivax* infections and to provide a cost benefice analysis of the possible introduction of these measures in a continuous program of malaria control.

The second objective will be reached when immigrant population will have access to Basic Primary Health Services in the localities to be settled. Special measures will be required for hydroelectric workers, at night shifts, working in riverside areas with large mosquito populations. Periodic health control, with parasitological examination, by sensitive molecular diagnostic procedures (PCR), will be essential. Night shift workers should be rotated and they should use chemoprophylaxis for *P.vivax* malaria for short period.
